# Van der Waals two-color infrared detection

**DOI:** 10.1038/s41377-022-00721-y

**Published:** 2022-02-01

**Authors:** Piotr Martyniuk, Antoni Rogalski

**Affiliations:** Institute of Applied Physics, Military University of Technology, 2 Kaliskiego Street, Warsaw, 00-908 Poland

**Keywords:** Optical materials and structures, Optical physics

*Light: Science & Applications* **11**, 6 (2022)

10.1038/s41377-021-00694-4

Two-color infrared detection technology realizes target recognition in a complex environment by using the multispectral characteristics of the target. In the last decade, several papers have announced the usefulness of the 2D materials for high operating temperature photodetectors covering infrared spectral regions. Researchers from Shanghai Institute of Technical Physics of Chinese Academy of Sciences, Huazhong University of Science and Technology, and Fudan University demonstrated an uncooled two-color infrared photodetector based on van der Waals heterojunction. This two-color photodetector can detect near-infrared and mid-wave infrared at the same time, and with ultra-low crosstalk, it realizes spectral blackbody detection with temporal and spatial coherence. Its room temperature operating ability greatly reduces the volume, weight, and power consumption of the detection components, and demonstrates the application prospects of van der Waals heterostructures in the miniaturized and intelligent photodetection systems.

